# In Silico Identification and Analysis of Proteins Containing the Phox Homology Phosphoinositide-Binding Domain in Kinetoplastea Protists: Evolutionary Conservation and Uniqueness of Phox-Homology-Domain-Containing Protein Architectures

**DOI:** 10.3390/ijms241411521

**Published:** 2023-07-15

**Authors:** Marina Petsana, Ahmed F. Roumia, Pantelis G. Bagos, Haralabia Boleti, Georgia G. Braliou

**Affiliations:** 1 Department of Computer Science and Biomedical Informatics, University of Thessaly, 2–4 Papasiopoulou Str., 35131 Lamia, Greece; marina.kpetsana@gmail.com (M.P.); af41555@gmail.com (A.F.R.); pbagos@compgen.org (P.G.B.); 2 Intracellular Parasitism Laboratory, Department of Microbiology, Hellenic Pasteur Institute, 11521 Athens, Greece; 3 Department of Agricultural Biochemistry, Faculty of Agriculture, Menoufia University, Shibin El-Kom 32514, Egypt

**Keywords:** phosphoinositides (PIs), Kinetoplastea protists, phylogeny, PX domain, BAR domain, sorting nexins, Pkinase domain, lipocalin domain

## Abstract

Kinetoplastea are free living and parasitic protists with unique features among Eukaryota. Pathogenic Kinetoplastea parasites (i.e., *Trypanosoma* and *Leishmania* spp.) undergo several developmental transitions essential for survival in their hosts. These transitions require membrane and cytoskeleton reorganizations that involve phosphoinositides (PIs). Phospholipids like PIs are key regulators of vital functions in all eukaryotes including signal transduction, protein transport and sorting, membrane trafficking, and cytoskeleton and membrane remodeling. A large repertoire of PI-metabolizing enzymes and PI-binding proteins/effectors carrying distinct PI-binding modules like the PX (phox homology) module could play significant roles in the life and virulence of pathogenic Kinetoplastea. The aim of this study was to retrieve the entire spectrum of Kinetoplastea protein sequences containing the PX module (PX-proteins), predict their structures, and identify in them evolutionary conserved and unique traits. Using a large array of bioinformatics tools, protein IDs from two searches (based on PFam’s pHMM for PX domain (PF00787)) were combined, aligned, and utilized for the construction of a new Kinetoplastea_PX pHMM. This three-step search retrieved 170 PX-protein sequences. Structural domain configuration analysis identified PX, Pkinase, Lipocalin_5, and Vps5/BAR3-WASP domains and clustered them into five distinct subfamilies. Phylogenetic tree and domain architecture analysis showed that some domain architectures exist in proteomes of all Kinetoplastea spp., while others are genus-specific. Finally, amino acid conservation logos of the Kinetoplastea spp. and *Homo sapiens* PX domains revealed high evolutionary conservation in residues forming the critical structural motifs for *Ptd*Ins3*P* recognition. This study highlights the PX-Pkinase domain architecture as unique within *Trypanosoma* spp. and forms the basis for a targeted functional analysis of Kinetoplastea PX-proteins as putative targets for a rational design of anti-parasitic drugs.

## 1. Introduction

Kinetoplastea are a class of flagellated protists which are members of the euglenozoa phylum. They comprise organisms with extremely diverse lifestyles and a range of features that distinguish them from other eukaryotes [1]. They are distinguished by the presence of kinetoplast, a DNA-containing region in their single large mitochondrion. This protozoan family, amongst other genera, includes the parasitic *Trypanosoma* and *Leishmania,* several species (spp.) of which are human pathogens that cause diseases with devastating health and economic consequences. Despite similarities in genomic organization, cellular structures, and life-cycle-associated morphological changes, various Kinetoplastea pathogens cause distinct vector-borne human diseases [2]. Understanding specific differences between Kinetoplastea pathogens in terms of gene sequence, protein function, and systems biology may lead to new insights for diagnostics, vaccines, and drug development to combat related diseases.

Kinetoplastea parasites undergo extensive cellular and developmental changes during their life cycle affecting protein trafficking, organelle formation and function as well as cell morphology. These diverse events are coordinated throughout the parasites’ developmental stages to maintain homeostasis and are essential for parasites’ survival in their vertebrate hosts and insect vectors [2,3,4]. Phospholipids and sphingolipids determine the membrane permeability and fluidity and have a profound impact on vesicular trafficking, nutrient acquisition via endocytosis, and cell differentiation, which involve extensive membrane remodeling/reorganization and macro autophagy processes linked to the differentiation events. In addition, the polyunsaturated fatty acid chains found in phosphatidyl choline (PC) could confer resistance to host-derived oxidants [5]. Moreover, the phosphatidylinositol (*Ptd*In)-based cellular regulatory network controls and coordinates numerous cellular processes taking place in developmental transitions and complex processes related to the adaptation of Kinetoplastea in hosts’ subcellular compartments [6]. While phospholipid and sphingolipid metabolism and the enzymes involved in it have become the focus of extensive studies in the pathogenic species of *Leismania* and *Trypanosomatids* (reviewed in [5,7,8,9])*,* the phosphatidylinositol (*Ptd*In)-based regulatory network has been poorly studied in these protozoans.

Numerous studies have linked the complexity of membrane lipids to signal transductions, organelle functions, physiological processes, and human diseases [10]). Specific protein–phospholipid interactions play key roles in many signal transduction pathways in all eukaryotic cells. Phosphoinositides (phosphatidylinositol phospholipids, (PIs)) are essential phospholipids located in the cytoplasmic leaflets of eukaryotic cell membranes. Despite contributing only a small fraction to the bulk of cellular phospholipids, they make remarkable contributions to practically all aspects critical for a cell’s life and death by exerting a wide variety of regulatory functions in the otherwise dynamic homeostatic organization of all eukaryotic cells, including lipid signaling, protein transport and sorting, membrane trafficking, and cytoskeleton and membrane remodeling [10,11]. They do so by recruiting cytoplasmic proteins/effectors or by interacting with cytoplasmic domains of membrane proteins at the membrane–cytoplasm interface to organize and mold organelle identity. A large repertoire of PI-binding proteins and PI-metabolizing enzymes accomplish these tasks by their localized functions and activities in concert with fluxes of PI metabolites as a response to environmental changes. This cohort of proteins and lipid metabolites constitutes the PI regulatory network. Due to the importance of the PI-based regulatory network for the eukaryotic cell’s life, the inhibition of key PI effectors or PI-metabolizing enzymes in eukaryotic pathogens could target and disrupt essential PI-regulated processes that allow pathogens to escape host defense. Thus, the pathogen-specific PI regulatory system provides protein sequences, structures, and functions that could be proven to be attractive drug targets against eukaryotic pathogens [12,13].

The physiological and regulatory roles of PIs are currently under extensive investigation both in mammals [14,15,16] and several unicellular eukaryotic organisms [12,17]. These functional lipids constitute a family of eight phospholipids consisting of *Ptd*In and its seven phosphorylated forms [11]. Site-specific phosphorylation on the 3, 4, and/or 5 hydroxyl positions of the inositol ring by a variety of lipid kinases leads to the generation of seven different compounds (i.e., *Ptd*Ins3*P* (PI3P), *Ptd*Ins4*P*(PI4P), *Ptd*Ins5*P*(PI5P), *Ptd*Ins(3,4)*P*_2_ (PI(3,4)P2), *Ptd*Ins(4,5)*P*_2_ (PI(4,5)P2, *Ptd*Ins(3,5)*P*_2_ (PI(3,5)P2), and *Ptd*Ins(3,4,5)*P*_3_ (PI(3,4,5)P3) [11,18,19]. At least five of these PIs (*Ptd*Ins4*P*, *Ptd*Ins(4,5)*P*_2_, *Ptd*Ins3*P*, and *Ptd*Ins(3,5)*P*_2_)) are almost ubiquitous in eukaryotes, with mostly similar [20] yet distinct and versatile functions [21]. The ubiquity of PIs in eukaryotes [11,22] suggests that some of their forms and functions in vesicular trafficking and cytoskeletal regulation probably appeared in an unidentified extinct ancestor of all eukaryotes, which may have been underway before early eukaryotes’ diversification [20,23,24,25,26].

To date, several distinct PI-binding protein modules have been identified and characterized, including PH (pleckstrin homology) [27,28]; ENTH (epsin amino-terminal homology) [29]); FYVE (named after the four cysteine-rich proteins in which it has been found (Fab 1, YotB, Vac1p, and EEA1) [30,31]; and PX (the phox homology domain) [32,33,34,35]. Such ‘cut and paste’ modules, found in a diverse array of multidomain proteins, mediate specificity in both partner recruitment and membrane binding to precise PIs embedded in the membrane bilayer and exert a multitude of signaling roles [36]. The binding of PIs to these domains is generally of low affinity and rapid reversibility, favoring thereby a system with high plasticity. These features enable signaling proteins involved in PI-mediated membrane association to undergo a sequence of random diffusions in the cytosol and dispersion within membranes along with binding and dissociation events rather than persistent associations with the membrane [37].

The PX domain was initially identified in *Homo sapiens*‘superoxide-generating neutrophil NADPH oxidase complex as a motif of approximately 130 residues within the p40phox and p47phox subunits [32]. The human genome encodes 49 proteins that possess the PX phosphoinositide-binding module with a wide range of functions, including roles in cell signaling and vesicle trafficking. The PX domain is conserved from yeast to human. A multiple alignment of representative PX domain sequences from eukaryotic proteins shows relatively little sequence conservation, although their structure appears to be highly conserved [33]. The structure of the human p40phox PX domain (PDB ID: 2DYB) and the structures of the PX domains from several other proteins have been solved by X-ray crystallography [36,38,39,40]. They consist of three alpha-helices and three beta-sheets. Evidence for the role of the PX domain as a PI(3)P-directed, membrane-targeting module has been provided by the analysis of human sorting nexin SNX3 [41].

Recent research has defined four distinct classes of human PX domains that either bind specifically to *Ptd*Ins3*P*, non-specifically to various di- and tri-phosphorylated phosphoinositides, bind to both *Ptd*Ins3*P* and other phosphoinositides, or associate with none of the lipids tested. A comprehensive evaluation of PX domain structures has revealed two distinct binding sites that explain these specificities, providing a basis for defining and predicting the functional membrane interactions of the entire PX domain protein family [38]. In general, PX domains ensure that proteins reach their appropriate intracellular membrane location by binding to the soluble inositol phosphate counterpart of their lipid binding partner in a PI-directed manner [42,43,44]. At the initiation of this study, 49 human and 14 yeast proteins containing the PX domain had been identified and reviewed (Uniprot v.2021_03) [38,45]. Although studies on lipid–protein interactions in humans and yeast constitute an emerging research field, little is known about these interactions in ancient eukaryotic human pathogens such as certain genera of Kinetoplastea protists.

Herein is presented a systematic in silico analysis performed in the Kinetoplastea proteomes existing in databases in order to identify in these protists protein sequences containing PX domain consensus sequences. The data mining approach employed was designed to retrieve all possible Kinetoplastea PX-domain-containing protein (PX-proteins) sequences from databases (deposited and reviewed) in order to investigate their conservation and structural architecture and assign to them known or yet unidentified structural and functional features. Moreover, the present study provides a thorough assessment of the conservation of PX motifs between Kinetoplastea and human PX-proteins. The reported findings can help discern specific features in the PX domain that could be responsible for distinct PI-binding specificities, which likely affect the subcellular targeting of each PX-protein linked to specific functional features.

## 2. Results

### 2.1. Retrieval and Identification of Kinetoplastea PX-Protein Sequences

**Figure 1:**
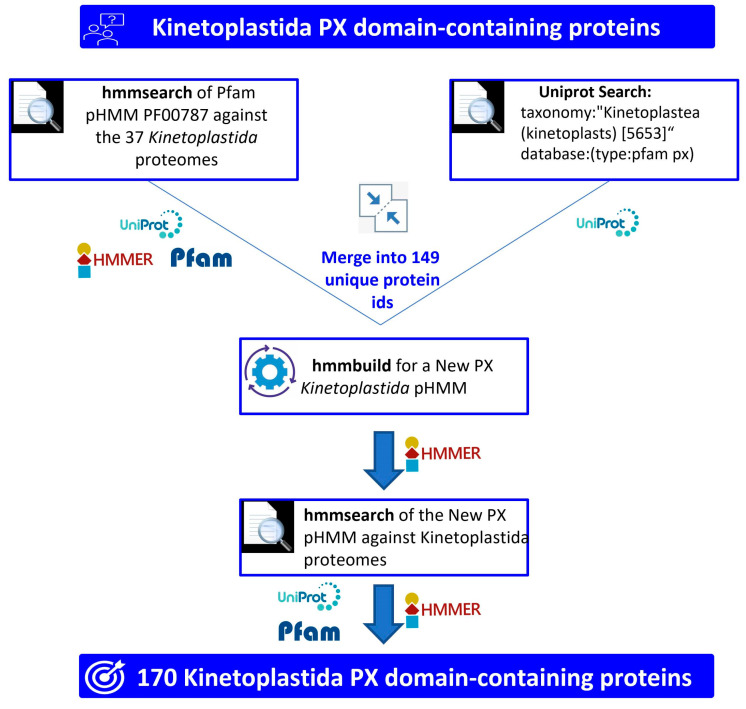
Methodological approach of the search for Kinetoplastea PX-domain-containing proteins (PX-proteins). Schematic diagram depicting the methodological steps followed for the retrieval and identification of the 170 Kinetoplastea PX-proteins.

The potential of sequence retrieval and analysis was used in this study to identify, classify according to structure, and infer potential functions to all Kinetoplastea PX domain-containing proteins (PX-proteins) registered in the databases. The retrieval methodology used herein consisted of a three-step approach. The first step involved the use of the Pfam’s pHMM for the PX domain (PF00787) to search against the 37 Kinetoplastea proteomes from 28 Kinetoplastea spp. found in Uniprot using the HMMER (v.3.3) software package. This protocol retrieved 137 PX-proteins (Figure 1). The second step was a search performed in the Uniprot database using as a cross-reference the PFam’s pHMM for the PX domain and the Kinetoplastea taxonomy ID. This query returned 143 PX-proteins. The proteins from the two above searches were merged into a new list comprising 149 unique protein IDs. Since 8 of these 149 IDs contained two PX domain sequences, the identified PX domain amino acid sequences were in total 157. These 157 sequences were aligned and utilized for the construction of a new pHMM (called hereafter Kinetoplastea_PX pHMM). Finally, the new Kinetoplastea_PX pHMM was used as a seed for a new search against the 37 Kinetoplastea proteomes downloaded from Uniprot using HMMER. The final number of proteins recovered with this third search step was 170 and included all the 149 proteins retrieved beforehand (Figure 1).

### 2.2. Evolutionary Analysis of the Kinetoplastea PX-Proteins

**Figure 2:**
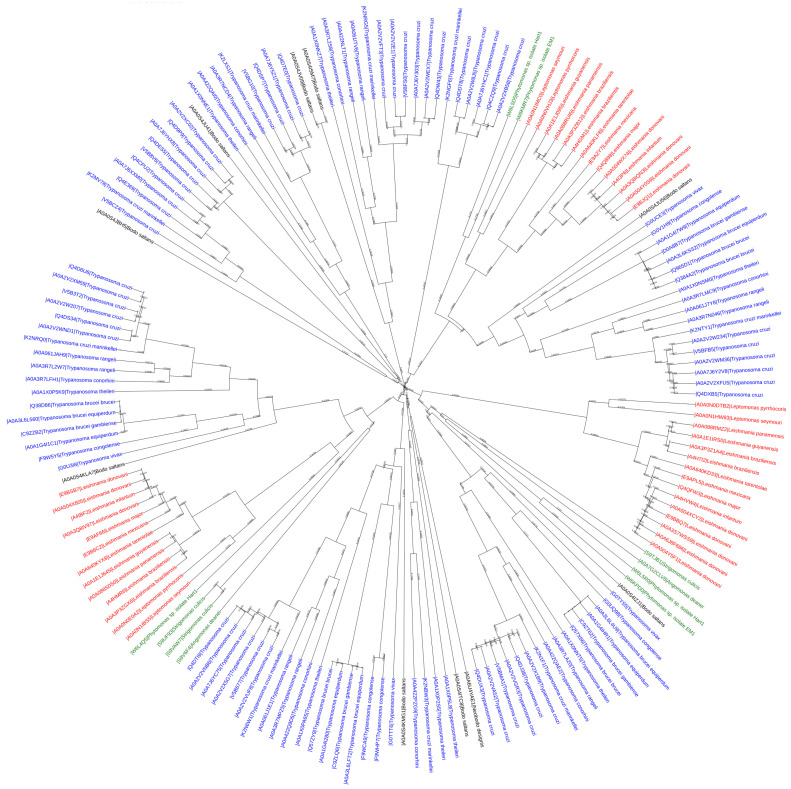
Phylogenetic tree of the 170 PX-proteins of Kinetoplastea. Different genera of Kinetoplastea organisms are presented with a different color. Blue: *Trypanosoma* spp.; red: *Leishmania* spp.; green: *Phytomonas, Angomonas*, and *Strigomonas* spp.; black: *Bodo* and *Neobodo* spp.

To explore the putative structural and functional relationships between the retrieved PX-proteins, their sequences were aligned and a sequence-based phylogenetic tree (neighbor-joining) illustrating their evolutionary relationship was constructed using the ClustalW and iTOL tools. As depicted in Figure 2, there are three coherent and well-supported major branches: the first one comprises proteins from only *Trypanosoma* and *Bodonidae* spp., while the other two include proteins from more Kinetoplastea genera (i.e., *Trypanosoma*, *Leishmaniae*, *Phytomonas*, *Angomonas*, *Strigomonas*, and *Bodonidae*). The above analysis verifies that *Bodonida* diversified first from the rest in the Trypanosomatida order. In addition, the fact that proteins from different species belong to the same clades suggests a broad structural divergence of Kinetoplastea PX-proteins, leading to a hypothesis that these proteins may contain additional structural and functional domains.

### 2.3. Kinetoplastea PX-Proteins Are Classified into Five Subfamilies: Structural Domain Architecture Analysis

**Figure 3:**
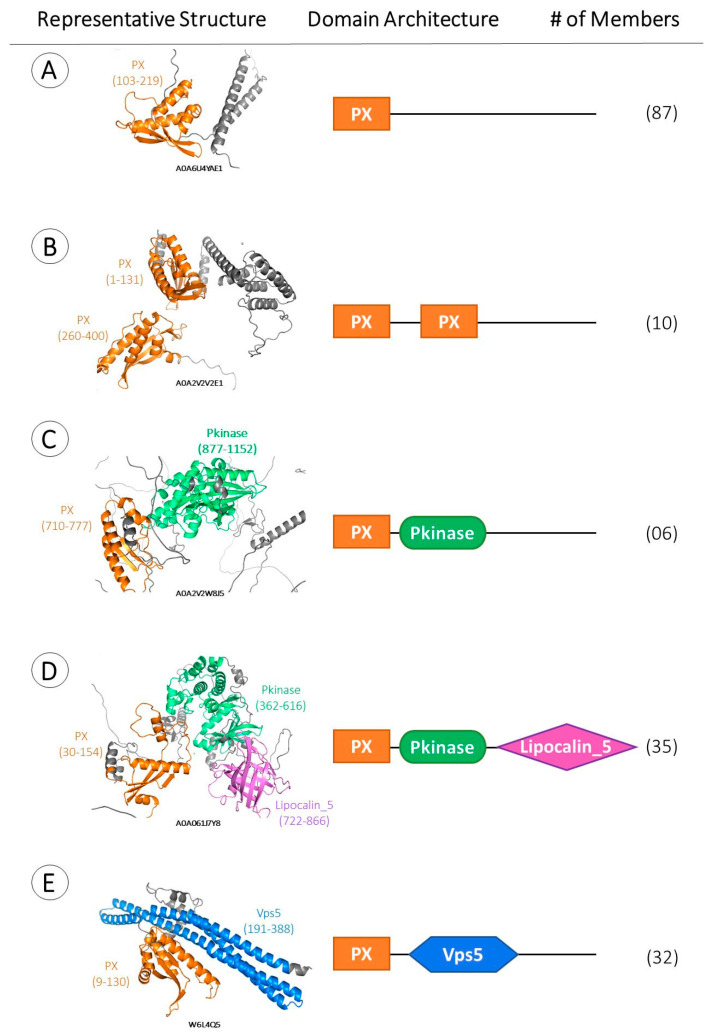
Schematic representation of the five Kinetoplastea PX-protein subfamilies along with AlphaFold structures of a representative of each family. Classification is based on differences in domain architecture as retrieved from databases. Different colors are used to represent each structural domain. PX: Orange (rectangular); Pkinase: green (oval); Lipocalin_5: magenta (rhombus); Vps5 (or BAR3-WASP): blue (stumped rhombus); unstructured or uncharacterized regions: grey. For reasons of comparison, areas of interest within AlphaFold structures are enlarged, and some unstructured regions may be omitted in representations of the (**B**–**D**) subfamily members. The numbers of protein members (#) in subfamilies (**A**–**E**) are shown in parentheses on the right.

To investigate the structural and functional relationship of the 170 Kinetoplastea PX-proteins retrieved as described above, *hmmscan* was implemented within the Pfam pHMM library and led to the retrieval of their configuration in terms of structural domains. These domains were subsequently verified manually in Intrepro [46]. Four different structural domains were identified: PX (Pfam profile ID: PF00787), Pkinase (PF00069), Lipocalin_5 (PF13924), and Vps5 (PF09325)/BAR3-WASP (PF10456) (Figure 3). It is important to emphasize that the terms Vps5 (PF09325) and BAR3-WASP (PF10456) are used interchangeably because the yeast Vps5 protein, a sorting nexin, has a characteristic coiled-coil domain in the carboxyl-terminal half, which is reported to carry a BAR-like (Bin/amphiphysin/Rvs) domain [40,46,47,48].

Based on their domain content and architecture, the 170 Kinetoplastea PX-proteins identified herein were classified into five distinct protein subfamilies (Figure 3). Subfamily A contains proteins with only one PX domain; subfamily B entails proteins with two PX domains; subfamily C has proteins with the PX and Pkinase domains; subfamily D has proteins with the PX, Pkinase, and Lipocalin_5 domains; and subfamily E comprises proteins with the PX and Vps5/BAR3-WASP domains. AlphaFold structures from representative members of each subfamily are shown in Figure 3.

### 2.4. Evolutionary and Structural Relatedness of the Kinetoplastea PX-Proteins

**Figure 4:**
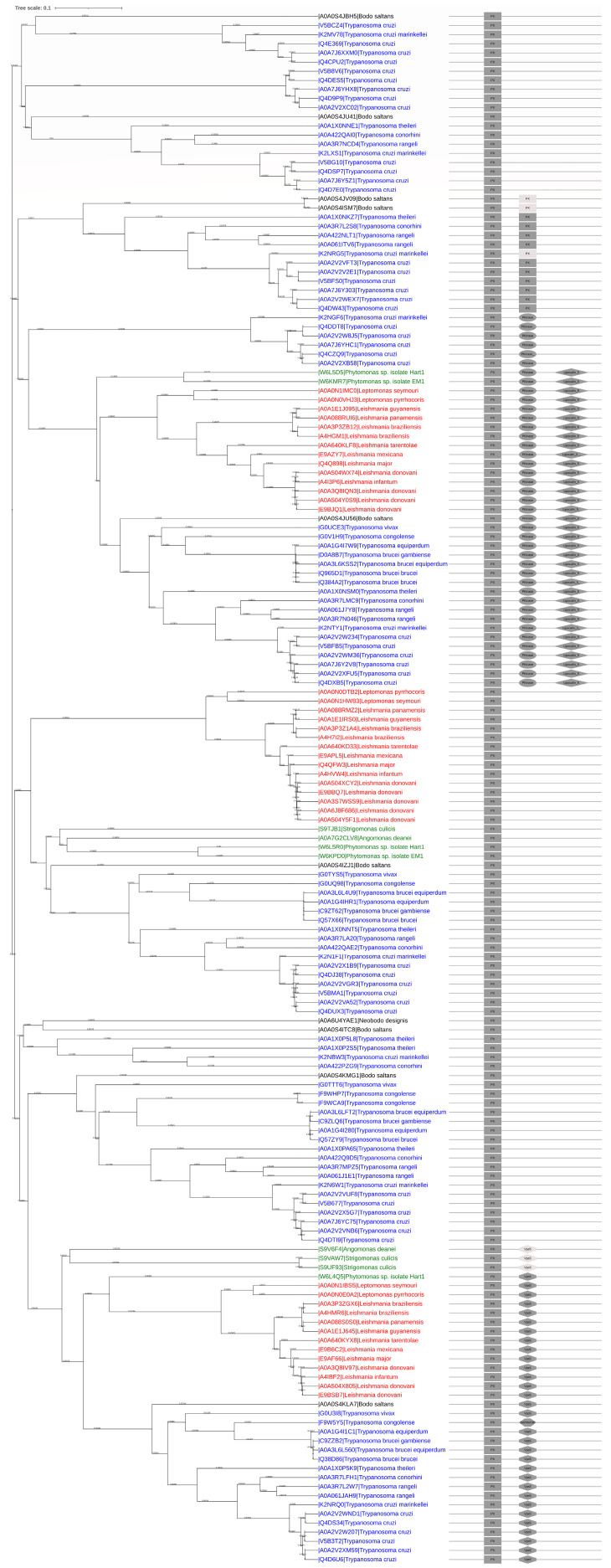
Evolutionary and structural analysis of the PX-protein subfamilies in Kinetoplastea. A sequence-based, neighbor-joining phylogenetic tree was constructed using iTOL. The domain architecture is added on the right of each protein. Each domain is represented by different shapes. PX: Rectangular; Pkinase: oval; Lipocalin_5: rhombus; Vps5 (or BAR3-WASP): stumped rhombus. Light grey symbols depict domains within proteins not reported by Uniport but predicted herein (see Section 2). Branch lengths depict evolutionary distance.

To further resolve the evolutionary and structural relationships between the 170 Kinetoplastea PX-proteins, our investigation was expanded to combine phylogenetic analysis with domain architecture relatedness. Thus, the schematic domain architecture of each protein, as depicted in Figure 3, was incorporated within the neighbor-joining phylogenetic tree representation near each PX-protein (Figure 4). A summary representation of the phylogenetic tree shown in Figure 4 with information about the organisms that contain PX-proteins with the five domain architectures and the number of proteins comprising each clade is shown in Supplementary Figure S1. Detailed information on domain architecture, length, and score values of the pHMMs scanned against the PX domain of each protein is presented in Supplementary Table S1.

Four main observations were highlighted by this side-by-side phylogenetic-tree-based evolutionary and domain architecture analysis: (a) all species have at least one protein with only one PX domain from subfamily A; (b) proteins that contain only one PX domain are the most abundant (87); (c) only *Trypanosoma* spp. contain proteins with two PX domains (subfamily B); (d) proteins that belong to the same phylogenetic clades and have evolutionary close distances tend to have the same domain architecture.

Moreover, the first branch contains proteins with only one PX domain that come solely from only two genera (*Trypanosoma* and *Bodo*) (Figure 2). The next branch consists of two major subclades, the first of which contains proteins with two PX domains. The other subclade contains proteins with PX and Pkinase domains, while a later diversification of this subclade entails proteins with an inserted Lipocalin_5 domain. The third branch consists of two major subclades, both of which comprise proteins with only one PX domain spanning all Kinetoplastea spp. However, the second subclade comprises proteins with either one PX domain or the PX and Vps5 (or BAR3-WASP) domains, the latter belonging to the same subclade. PX-Vps5 proteins are also found in all Kinetoplastea. As seen in the phylogenetic trees (Figure 2 and Figure 4), the earliest appearance of all additional structural and functional domains is that of Vps5, suggesting a vital role of this domain in the survival of all Kinetoplastea. Interestingly, regarding three proteins (i.e., tr|K2NRG5|*Trypanosoma cruzi marinkellei*, tr|A0A0S4JV09|*Bodo saltans*, and tr|A0A0S4ISM7|*Bodo saltans*) (Figure 4), although they belong to the same clade as B subfamily proteins (PX-PX architecture), they are reported by Uniport and Pfam to contain only one PX domain. Given the evolutionary and structural relationships of the 170 proteins described in Figure 4, this phylogenetic tree analysis can be used to predict structures of proteins evolutionary closely related. To test this hypothesis, the sequences of the above proteins were aligned with the rest of the proteins of the same subclade. Multiple sequence alignment (MSA) revealed high homology scores within both PX domains (especially in the core *Ptd*Ins3*P* binding motifs; Supplementary Figure S2). Thus, our approach classified them correctly as members of the subfamily B proteins. Similarly, the proteins tr|S9UF93|*Strigomonas culicis*, tr|S9VAW7|*Strigomonas culicis*, and tr|S9V6F4|*Angomonas deanei*, classified in the clade entailing the PX-Vps5 domain architecture, were reported in Uniprot to have a unique PX domain. Again, the sequence alignment of all proteins belonging to the same clade revealed sequence similarity in both the PX and Vps5 domains (Supplementary Figure S3), highlighting further the validity of the present sequence-based, neighbor-joining phylogenetic tree analysis to predict protein domain architecture.

### 2.5. Kinetoplastea PΧ-Protein Subfamilies Can Be Either Species-Specific or Ubiquitous

**Figure 5:**
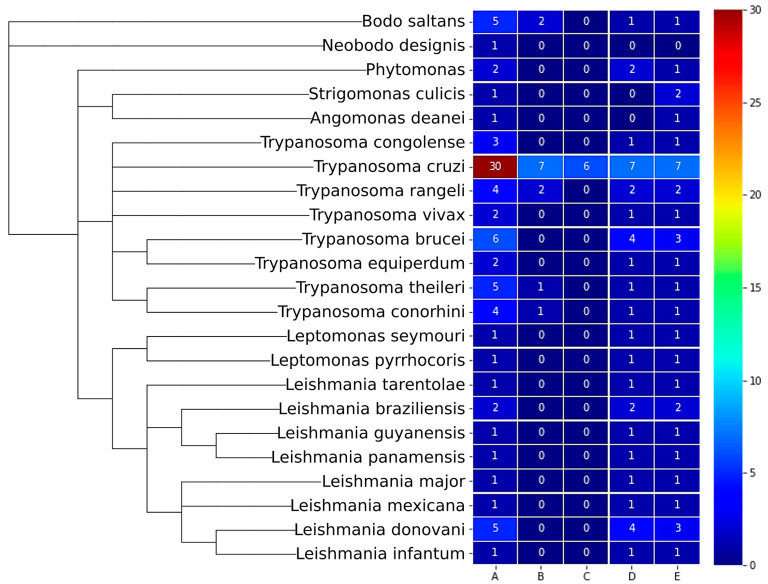
Distribution of PX-proteins in Kinetoplastea based on domain architecture subfamilies. Phylogenetic tree of the Kinetoplastea organisms (**left**) utilizing NCBI Taxonomy browser and iTOL visualization tool in combination with Heatmap representation (**right**) of PX-protein subfamilies’ distribution of the 170 identified Kinetoplastea PX-proteins. Adjustments from predictions made by domain architecture analysis have been implemented. Heatmap color scale; dark blue: zero proteins; red: ≥30 proteins. Numbers inside the cells indicate the number of proteins from each protein subfamily identified in each organism. A, B, C, D, E at the bottom of the table represent the five subfamilies of domain architecture.

The next question investigated in this study was whether, within the Kinetoplastea genera, all species and subspecies express proteins from all subfamilies or whether some of these proteins are specific for only one or few species. In this direction, the distribution of these 170 proteins among the 23 Kinetoplastea spp., classified into different subfamilies, was analyzed according to their domain architecture, and a heatmap was created in order to visualize the results. As shown in Figure 5, most proteins belong to subfamily A (one PX domain), while the smallest subfamilies comprise proteins that contain either two PX domains or the PX and Pkinase domains (13 and 6 members, respectively).

*Trypanosoma cruzi* is the organism with the highest number of PX-proteins, while *Neobodo designis* is underrepresented in this heatmap. All Kinetoplastea spp. for which were retrieved sequences from the databases, were found to contain one protein from subfamily A. Intriguingly, almost all species have proteins with the PX-Vps5 (subfamily E) and PX-Pkinase-Lipocalin_5 domains (subfamily D), suggesting that these domain combinations may be fundamental for the survival of Kinetoplastea protists. Considering the above assumption, it emerges as extremely possible that *Strigomonas culicis* and *Angomonas deanei* do contain proteins with domain architectures of subfamily D, while *Neobodo designis* may contain proteins that are not yet identified from both subfamilies D (PX-Pkinase-Lipocalin_5) and E (PX-Vps5/). Similarly, since four out of eight *Trypanosoma* spp. have proteins of subfamily B (PX-PX), it could be postulated that all *Trypanosoma* spp. have PX-PX-containing proteins. Moreover, since *Trypanosoma cruzi* contains proteins belonging to subfamily C, it is also possible that all *Trypanosoma* spp. have proteins with domain architectures from subfamily C (PX-Pkinase). Whether the proteomes of these protists encompass such proteins remains to be investigated. The existence of such predicted proteins with additional domains is further supported by the fact that, while the members of subfamilies B (PX-PX), C (PX-Pkinase), and E (PX-Vps5) have almost the same number of residues, subfamily D contains three clear subgroups of proteins, as classified by their length (i.e., 872/1165/1305 aa on average (Supplementary Table S1)). Even more interestingly, within subfamily A (only PX), there is a high divergence in the number of residues of each protein, suggesting the existence of heterogeneous proteins not only in length but in structure and function as well.

### 2.6. Sequence Conservation Analysis of the Kinetoplastea PX Domains

**Figure 6:**
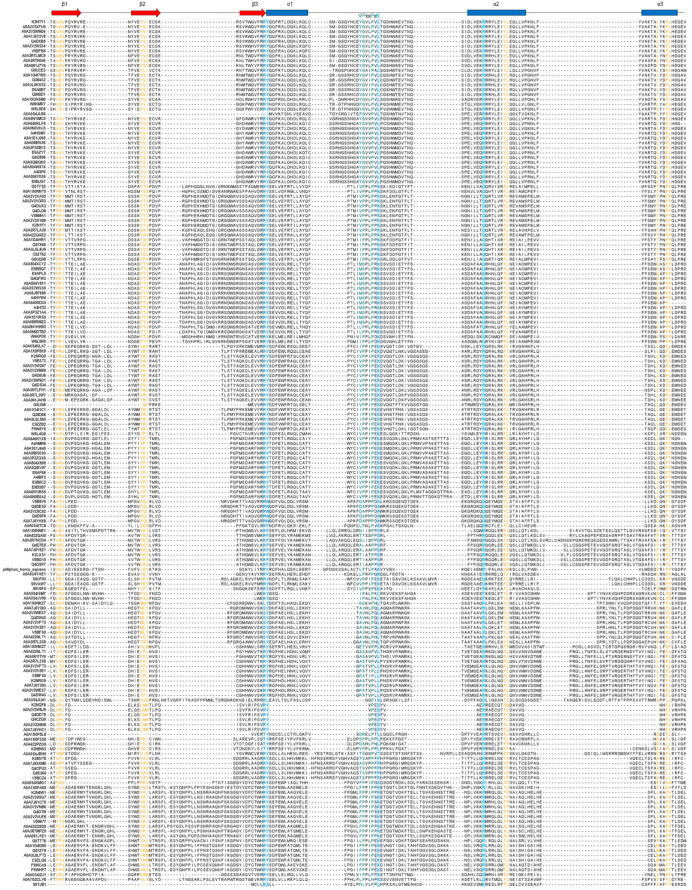
Secondary-structure-guided sequence alignment of the Kinetoplastea PX domains. ClustalW-based sequence alignment of the 180 Kinetoplastea PX domains and visualization with Jalview. A secondary structure prediction of the proteins is depicted at the top based on secondary structure elements from the crystal structure of the PX domain of the human p40phox used as the reference sequence according to Chandra et al. [38]. The accuracy of sequence alignment was additionally refined manually to attain more precise structural comparisons of the PX domain sequences. The red arrows above the multiple sequence alignments (MSA) represent beta-sheets and the blue rectangles alpha-helices. The four residues (RYKR) critical for *Ptd*Ins3*P* recognition and binding are depicted with cyan letters. The binding pocket for a canonical lipid *Ptd*Ins3*P*, the polyproline loop containing the ΨPxxPxK sequence motif (Ψ = hydrophobic side chain), is shown in green (PP)/cyan (K).

Since ten out of the 170 Kinetoplastea PX-proteins retrieved in this search contained two PX domains, the total number of PX domain sequences was 180. Consequently, the multiple sequence alignment (MSA) of these 180 PX (isolated) sequences was performed using ClustalW. Figure 6 presents the visualization of this MSA with Jalview. A secondary structure prediction was also carried out using as reference the secondary structure elements deduced from the crystal structure of the human p40phox PX domain [38]. As shown in Figure 6, the four residues RYKR (cyan color) that, according to Chandra et al. [38], comprise the critical motif for *Ptd*Ins3*P* recognition and binding are highly conserved. In addition, the polyproline binding loop, also called the PPK loop (containing a ΨPxxPxK sequence motif, where Ψ = hydrophobic side chain), was also relatively conserved. Conserved were also found certain residues from the β3 strand and the α1 helix that form the binding pocket for the canonical lipid *Ptd*Ins3*P* headgroup (Figure 6). Finally, conservation was also observed for certain residues in the alpha 2 and alpha 3 helices and to a lower extent in the beta1 and beta 2 sheets (Figure 6).

**Figure 7:**
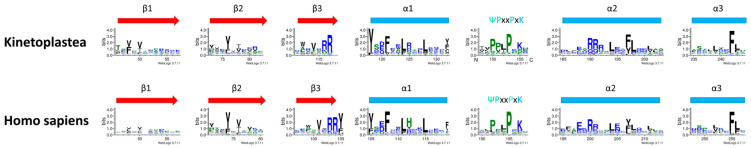
Amino acid conservation logos of the PX domains of Kinetoplastea spp. and *Homo sapiens*. Amino acid conservation logos were constructed from 180 Kinetoplastea PX domain sequences (**upper** panel) and 183 *Homo sapiens* PX domain sequences (**lower** panel). All sequences were retrieved from Uniprot. Red arrows symbolize the β-sheet regions, and blue rectangle boxes symbolize the α-helices. The higher the residue letter size, the higher the conservation.

The extremely high conservation revealed in certain regions of the PX domain triggered us to investigate how critical motifs within this domain are conserved between evolutionary distant species. Given that the PX-proteins have been extensively studied in *Homo sapiens*, which is one of the main host organisms for the pathogenic Kinetoplastea, we were prompted to investigate the structurally and, conceivably, functionally conserved PX domain elements between Kinetoplastea and *Homo sapiens*. For this, conservation logos of Kinetoplastea PX motifs were constructed from the amino acid sequences of the 180 Kinetoplastea PX domain sequences. In addition, the conservation logos of the human PX motifs of 183 PX domain sequences from *Homo sapiens* retrieved from Uniprot were also constructed using the Praline tool in combination with manual refinement (Supplementary Figure S4) subjected to the Weblogo3 tool (Figure 7). A comparison of the three alpha-helices and the three beta-sheets of the Kinetoplastea PX domains with those of *Homo sapiens* showed high amino acid conservation. The beta 1 sheet sequence showed the least conservation in all species. Similarly to results from *Homo sapiens* PX-proteins [29], the region between the alpha 1 and alpha 2 helices, although unstructured, was found to be highly conserved in Kinetoplastea as well since it constitutes the core loop of the PI binding site. It therefore seems that the PX domain is a structural domain with a specific function that has been well conserved during the evolution of eukaryotes, and possible deviations in it may serve membrane binding specificity.

## 3. Discussion

Combating vector-borne, neglected tropical diseases suffers from limited chemotherapeutic agents with severe side effects, against which protozoan parasites often develop resistance [49,50]. With the advent of high-throughput technologies, new perspectives have opened towards the design or identification of new drugs. The emerging challenges for the discovery of novel drugs for diseases caused by pathogenic Kinetoplastea lie in the acknowledgment and exploitation of the species- and even strain-specific features of the parasites such as virulence power, specificity of cell invasion, defense against the host immune response, intracellular growth rate, parasitemia, tissue tropism, and resistance and specificity to drugs (reviewed in [51]). Enzymes involved in the above pathways and features of the parasites have been identified as drug targets in *Trypanosoma* and *Leishmania* spp. For example, a vinyl sulfone derivative (K177) is a newly discovered drug against a cysteine protease of *T. cruzi* (cruzipain 1). Cruzipain exhibits high homology to the catalytic N-terminal domain of human cathepsin B and L but contains a unique glycosylated C-terminal domain on which K177 can exert its anti-cruzipain activity [51,52].

With a chemogenetic approach, (i.e., gene knockout), enzymes involved in the inositol phosphate metabolic pathway, and which are essential for the parasite’s life cycle, have been identified as potential drug targets against Kinetoplastea parasites [53]. Phosphoinositides (PIs) and inositol phosphates (IPs) play pivotal roles in the complex network that is signaling and regulating processes important for protozoan pathogens’ developmental stages [6]. Some of these processes, alternating between mammalian host and insect vector, are considered to help pathogens escape host defense mechanisms [7,26].

PIs and proteins that bind to PIs are found in all eukaryotes and contribute to protein networks orchestrating the PI-based regulation of vital cell functions [49]. PI-binding proteins can be sorting nexins, phospholipases, kinases, etc. They contain certain PI binding motifs, of which one is PX, and have a multimodular structure [36]. The absence of studies on the role of Kinetoplastea PX-proteins that bind to PIs, the finding that the inositol phosphate metabolic pathway is druggable [53], and the growing availability of sequenced Kinetoplastea spp. genomes prompted us to carry out this in silico study focusing on the identification, phylogenetic analysis, and prediction of the functionality of all PX-proteins in Kinetoplastea protists for the first time.

Using a Pfam pHMM profile of the PX domain and a comprehensive three-step strategy (entailing the construction of a Kinetoplastea_PX pHMM), all 170 Kinetoplastea PX-domain-containing protein sequences (PX-proteins) registered in databases were retrieved; none of these proteins has been functionally characterized before. MSA guided a reconstruction of their evolutionary history and the investigation of their structural domain composition. Phylogenetic analysis depicted a broad distribution of various Kinetoplastea spp. Among branches of PX-proteins, which were indicative of greater sequence conservation within clades. It has been reported that human and yeast sorting nexins contain additional protein–protein and protein–PI interaction motifs (i.e., BAR/Vps5, GAP, PH, SH3, PDZ, BAR, FERM, MT, kinesin) [36,54]. These additional domains can be either an alternative PI interaction motif (perhaps specific to different forms of phosphorylated PIs) or protein–protein interaction domains that convey a multitude of functions [36]. Accordingly, we hypothesized that Kinetoplastea PX-proteins belonging to the same clade could possess additional conserved sequences corresponding to structural motifs.

The phylogenetic analysis of the entire length of the 170 PX-proteins revealed that proteins of the same clade presented not only higher homology but the same domain architecture as well. We show that the Kinetoplastea PX-proteins can be classified intο five subfamilies with one PX domain (A) and double PX (PX-PX) (B), PX-Pkinase (C), PX-Pkinase-Lipocalin_5 (D), and PX-Vps5 domains (E). Deviations from the apparent tendency of proteins classified in the same clade to contain the same domain architectures were initially taken as exceptions (Figure 4); however, successive MSAs revealed sequence conservation within subclades, thereby validating the present phylogenetic and domain architecture analysis as a structure prediction tool.

Extensive database searches led to the important finding that the PX-Pkinase-Lipocalin_5 architecture was not present in any other eukaryotic species apart from Kinetoplastea Protista (Supplementary Table S2), thus raising the possibility for these proteins to be used as Kinetoplastea-specific drug targets should their functions be proven essential for the parasites’ survival within the mammalian host. Similarly, extensive Uniprot searches and searches of related databases showed that PX-Pkinase proteins are not reported as such in any organism but rather as Pkinase-containing proteins. This observation suggests that proteins with such domain architectures have not been studied yet or that PX-Pkinase proteins are unique to Kinetoplastea, a hypothesis that demands further investigation. Surprisingly, PX-PX-containing proteins were found not only in unicellular organisms (parasites or free-living organisms) but in few higher eukaryotic organisms as well, such as plants (*Actinidia chinensis* var. *chinensis*), insects (*Rhipicephalus pulchellus*), birds (*Zosterops borbonicus*), and mammals (*Macaca fascicularis*) (Supplementary Table S3).

Importantly, the two PX domains identified in the members of the B subfamily have considerable amino acid differences. They are not the exact copies of the same PX domain sequence. It has been proposed that human PX domains comprise four distinct classes with varying PI-binding specificities: (a) a high specificity for *Ptd*PI(3)*P*; (b) a lower specificity for di- and tri-phosphorylated PIs; (c) binding to *Ptd*PI(3)*P* and various other PIs; (d) no binding to tested PIs [38]. A comprehensive evaluation of PX domain sequences, performed herein revealed important differences between the two PX domains in PX-PX-proteins (Supplementary Figure S2). This finding supports the assumption that divergence in the PX domain sequence could regulate the PI binding specificity of different proteins, providing a basis for defining and predicting the functional membrane interactions of the entire PX-protein family [36,38].

The conservation detected in the residues of the core PI binding region across the diverse members of the Kinetoplastea PX-protein subfamilies underline their importance in the overall structure and function of the PX domain. The core PI binding region spans the beta 3 sheet and the alpha 1 and alpha 2 helices, including PI binding residues within the core polyproline loop (a highly conserved ΨPxxPxK sequence (Ψ = large hydrophobic amino acids V, I, L, and M) (Figure 6 and Figure 7). This conserved motif is located within a highly extended helix-turn-helix structure formed by certain residues of the beta 3 strand, the PPK loop, and the alpha 2 helix. Thus, the findings presented herein highlight that this binding motif has not diversified significantly along the evolution of eukaryotes from Kinetoplastea to *Homo sapiens* (Figure 7). However, deviations were observed in the core PPK loop in all alpha-helices and beta-sheets. This is indicative of extensive evolutionary genetic events, in contrast to the more conserved human sequence logo.

Taken together, our findings suggest the existence of a multigene family of PX-proteins in Kinetoplastea. The present study shows that, although the PX domain is a structural and functional feature of many proteins within eukaryotes, three combinations of PX and other domains (PX-PX, PX-Pkinase, and PX-Pkinase-Lipocalin_5) seem to be unique to Kinetoplastea spp. Additionally, PX proteins and PX-Vps5 are found in many eukaryotes, including humans and yeast [44]. We propose that these three subfamilies may constitute potential Kinetoplastea-specific drug targets. In this direction, experimental studies are required to uncover the functional roles of these proteins, i.e., to identify biological substrates and effector proteins and investigate expression patterns, biological importance, roles in virulence, life cycle transitions, and resistance to host immune response, perhaps via targeted mutations and knockout experiments. Due to this first description of these Kinetoplastea PX-protein subfamilies with domains not yet found in other eukaryotes, their many roles are unknown. In a second step, molecular dynamics simulations, cheminformatics, and docking approaches can be recruited to identify potential drugs against certain PX-proteins as applied for other parasitic proteins [52]. For example, the two PX domains of B subfamily members (found only in *Trypanosoma*), despite having the same folding, they differ in some residues in their catalytic domain. This may dictate substrate preferences, helping thereby in the development of selective inhibitors for these proteins.

Used as a predictive tool, the above structural domain architecture and distribution analysis could lead to several conclusions concerning evolutionary events and protein functions. First, all Kinetoplastea spp. analyzed in this study contained members from the A, D, and E subfamilies, i.e., proteins containing the PX (A), PX-Pkinase-Lipocalin_5 (D), and PX-Vps5 domains (E). This may indicate a vital function of these proteins in all Kinetoplastea spp. In addition, the fact that the PX-Pkinase-Lipocalin_5 subfamily is found only in Kinetoplastea, and in no other eukaryotes, denotes its involvement in important genera-restricted functions. Second, the fact that *Bodo saltans* has proteins of subfamilies B, D, and E, while no such proteins were identified in *Neobodo designis*, may reflect the limited number of studies performed with these two marine free-living protists [1,54]. However, certain differences are expected since these free-living protists may have evolved differently, and they thus lack—or fail to express—other proteins in subcellular structures that may better support the non-parasitic lifestyle of these flagellates [55]. The fact that the majority of the retrieved proteins (108) were from *Trypanosoma* spp. can be attributed to *Trypanosoma* being the earliest (first described in 1886 [56]) and most extensively studied genus from the Kinetoplastea class compared to other genera, e.g., *Leishmania* (first described in 1904 [57]), for which only 36 PX-proteins were retrieved from the databases. Third, the PX-PX domain architecture obviously represents the product of a duplication event early in evolution restricted to *Trypanosoma* and *Bodo saltans* spp., coming perhaps from a common ancestor that gave rise to this specific subfamily (Figure 4). Gene duplication is a procedure known to occur for other proteins as well [58,59,60], and it is crucial for the appearance of novel genes. It is possible that the generation of new molecular structures or the elimination of others is part of the adaptation process adopted by these parasites as they evolved throughout the evolutionary tree of life within the diversity of the encountered microenvironments. Overall, it seems that, apart from duplication, other successive rounds of translocation and divergence events have probably led to the appearance of PX-protein subfamilies in all Kinetoplastea.

The present analysis cannot rule out the possibility that other structural motifs exist in each of the 170 Kinetoplastea proteins identified in this search. This is supported by the fact that, while PX-PX (subfamily B), PX-Pkinase (subfamily C), and PX-Vps5/BAR proteins (subfamily E) present a unique protein length distribution of about 590 aa, 1178 aa, and 419 aa, respectively, (apart from two proteins in specific strains) (Supplementary Table S1), subfamily D comprises three protein groups on the basis of aa length (i.e., mean length 872 aa for *Trypanosoma*, 1165 aa for *Phytomonas* and *Bodo saltans*, and 1305 aa for *Leishmania* genera) (Supplementary Table S1). Moreover, subfamily A includes a wide range of protein lengths, supporting the existence of many other, not yet identified, structural domains. Taken together, the above highlight the need for further structural investigation of the 170 Kinetoplastea PX-proteins to explore their modular structure; the existence of other domains such as SH3, PDZ FERM, and PH; and other structural motifs described already in human and yeast sorting nexins [44].

PIs and their metabolism in the host organisms are critically involved in the interactions of intracellular pathogens with their host cells. Several bacteria pathogens target PI metabolism at the plasma membrane of the host cells, thus modulating their uptake and anti-apoptotic signaling pathways. Employing this strategy, amongst other examples, *Shigella flexneri* directly injects a PI-modifying effector protein, while *Listeria monocytogenes* exploits PI metabolism indirectly by binding to transmembrane receptors [61,62,63,64]. Moreover, an intricate competition exists between intracellular pathogens and host cells in the control of cellular trafficking regulated by sorting nexins (SNXs; PX-containing PI-binding proteins). Several intracellular bacteria pathogens such as *Salmonella Typhimurium, Chlamydia trachomatis*, *and Legionella pneumophila* hijack PI binding as part of the mechanisms used to support their intracellular survival [65,66,67,68,69]. Similarly to pathogenic bacteria, eukaryotic pathogens, such as the oomycete *Phytophthora infestans* and the apicomplexan *Plasmodium falciparum*, appear to utilize PI(3)P at the host plasma membrane and the parasite endoplasmic reticulum (ER) lumen to modulate, respectively, endocytic and exocytic trafficking pathways for both secretion and pathogenesis [22,70]. Since most of the Trypanosomatidae members of Kintoplastea are obligate intracellular pathogens (i.e., *Trypanosoma* spp. and *Leishmania* spp.) [71], the identification and study of PI-interacting proteins involved either in the signal transduction pathways mediated by PIs or in PI-metabolism could highlight potential anti-parasitic drug targets given the extremely important role of PI-interacting proteins in the cellular processes fundamental to eukaryotic life.

## 4. Conclusions

*Trypanosoma* and *Leishmania* spp. pathogenic Kinetoplastea protozoa are responsible for some of the most severe neglected tropical diseases when transmitted by arthropod vectors to humans. These diseases constitute a global health burden, although they are more endemic to countries around the equator [72]. As existing treatments present several problems, and since there is a lack of an effective vaccine for humans, there is an urgent need for understanding the virulence mechanisms employed by the pathogenic strains of *Trypanosoma* and *Leishmania* in order to develop novel treatments [73]. In this study, the power of bioinformatics’ methodological strategies was employed to gain insight into evolutionary conserved PI-binding PX-proteins in Kinetoplastea and investigate sequence and structural features underpinning functions which, after experimental validation, may prove to be pivotal and unique for the pathogenic protozoans’ life cycle. Our results lay the foundation for the design of experimental approaches aiming to elucidate the biochemical and cellular functions of the herein discovered Kinetoplastea PX-proteins in a targeted manner. The proteins proven to be essential for the pathogens’ life cycle in mammalian hosts and simultaneously unique to pathogens or structurally very different to their mammalian homologues will constitute targets for a rational design of anti-parasitic interventions. Moreover, the experimental protocol followed in this study could be applied for similar studies in other protozoan pathogens to identify new members of the PX-protein family not only for translational applications but also for important evolutionary studies.

## 5. Materials and Methods

### 5.1. Data Mining, Retrieval, and Identification of PX-Domain-Containing Proteins in Kinetoplastea

Both Pfam (v.34.0), a repertoire of 19,179 protein families along with their respective profile hidden Markov models (pHMMs) [40], and Uniprot (v. 2021_03), the most comprehensive and freely accessible database of proteins [74], were used to retrieve all possible proteins containing PX domain sequences in Kinetoplastea.

As a first step, the PX domain pHMM (pfam profile ID: PF00787) was downloaded from Pfam, and hmmsearch (command) of the HMMER (v.3.3) packet in Linux environment [40,74,75] was executed against the Kinetoplastea proteomes retrieved from Uniprot (v.2021_03). Secondly, a search was performed in Uniprot utilizing the following query: “taxonomy: “Kinetoplastea (kinetoplasts) [53,56]”; database: (type:pfam px)” (accessed on 10 December 2022). Subsequently, the unique retrieved protein members from both searches were aligned using ClustalW [76,77] and then refined using MUSCLE [78]. Pfam (v.34.0) and Interpro (v.86.0), the protein domain and classification databases, were utilized to isolate only the PX domain sequences from the retrieved proteins, and a new PX domain pHMM (Kinetoplastea_PX pHMM) was built using the hmmbuild command of HMMER. Finally, using the Kinetoplastea_PX pHMM and the hmmsearch command of HMMER, a new search was performed against the Kinetoplastea proteomes to retrieve extra protein members that could have been missed in the previous search. A pHMM score value of 15 was used as a threshold for protein retrieval, which corresponded to the lowest score of true positive members. The heatmap was created using the Python (3.7) programming language and the Matplotlib library.

### 5.2. Identification of PX-Domain-Containing Proteins in Homo sapiens

The PX-domain-containing proteins of *Homo sapiens* were retrieved from Uniprot using the query: “taxonomy: “Homo sapiens (Human) [9606]”; database: (type:pfam px)” (accessed on 10 December 2022).

### 5.3. Determination of Domain Architecture and Construction of Motifs

All retrieved PX-proteins from Kinetoplastea were searched using *hmmscan* within the Pfam pHMM library to identify all possible known protein domains as well as their domain architectures. Subsequently, all domain architectures were manually confirmed using Uniprot. Pfam, and Interpro to assess boundaries and isolate only the PX, Pkinase, Lipocalin_5, and Vps5 domain sequences from the retrieved proteins. Consequently, the PX domains were aligned using ClustalW and, after manual curation, were submitted to Weblogo3 [79] with default options to generate amino acid conservation logos of PX domain alpha-helices and beta-sheets.

### 5.4. Phylogenetic Analysis

To investigate the evolutionary relationship of Kinetoplastea PX-proteins, the retrieved sequences were aligned using ClustalW. The resulting multiple sequence alignment was used to reconstruct and visualize a distance-based phylogenetic tree employing the neighbor-joining method implemented by iTOL [80]. Taxonomy tree data were retrieved from the Natural Center for Biotechnology Information (NCBI Taxonomy) [81].

## Data Availability

All relevant data are within the manuscript and its Supporting Information files.

## Supplementary Materials

The following supporting information can be downloaded at: https://www.mdpi.com/article/10.3390/ijms241411521/s1.
