# Supplementary material for: In Silico Identification and Analysis of Proteins Containing the Phox Homology Phosphoinositide-Binding Domain in Kinetoplastea Protists: Evolutionary Conservation and Uniqueness of Phox-Homology-Domain-Containing Protein Architectures
Source: Int J Mol Sci. 2023 Jul 15;24(14):11521. doi: 10.3390/ijms241411521 (PMC10380299; doi:10.3390/ijms241411521)
Supplement: Supplementary file 1 [file ijms-24-11521-s001.zip › Supplermentary figure legends.pdf]

Supplementary Figure legends

Supplementary Figure S1. Schematic, summary representation of the phylogenetic tree shown in Figure 4. The organisms, the domain architectures and the number of proteins belonging to each clade which possess the indicated domain architecture are presented. Organisms are indicated with colored dots. Blue: *Trypanosoma* spp; black: *Bodonidae*; red: *Leishmaniae*; green: *Angomonas*, *Strigomonas* and *Phytomonas* spp. Numbers in brackets depict the number of proteins of each clade with the indicated domain architecture.

Supplementary Figure S2. Multiple Sequence Alignment (MSA) of PX-PX Kinetoplastea proteins (B subfamily) using ClustalW. A0A0S4JV09, A0A0S4ISM7, and K2NRG5 proteins are predicted by this study to belong to subfamily B. The four residues (RYKR) critical for *Ptd*Ins3*P* recognition and binding are bracketed within red boxes. The PX domain from the human p40phox NADPH subunit is used as reference.

Supplementary Figure S3. Multiple Sequence Alignment (MSA) of PX-VPS5 Kinetoplastea proteins (E subfamily) using ClustalW. S9V6F4, S9VAW7, and S9UF93 proteins are the ones predicted by this study to belong to subfamily E. The four residues (RYKR) critical for *Ptd*Ins3*P* recognition and binding are bracketed within red boxes. The Vps5 domain sequence from human SNX1 is used as reference.

Supplementary Figure S4. Secondary structure-guided sequence alignment of the Kinetoplastea PX domains. ClustalW-based sequence alignment of the 183 human PX domains and visualization with Jalview. A secondary structure prediction of the proteins is depicted at the top based on secondary structure elements revealed in the crystal structure of the PX domain of the human p40phox NADPH subunit used as reference sequence according to Chandra et al. [32]. The accuracy of sequence alignment was additionally refined manually according to Chandra et al. Red arrows above the MSA represent beta-sheets and blue rectangles represent alpha-helices. The four residues (RYKR) critical for *Ptd*Ins3*P* recognition and binding are depicted with cyan letters. The binding pocket for a canonical lipid *Ptd*Ins3*P*, the polyproline loop containing the Ψ**P**xx**P**x**K** sequence motif (Ψ = hydrophobic side chain), is shown in green (PP) /cyan (K).
