# Supplementary figures and images for: In Silico Identification and Analysis of Proteins Containing the Phox Homology Phosphoinositide-Binding Domain in Kinetoplastea Protists: Evolutionary Conservation and Uniqueness of Phox-Homology-Domain-Containing Protein Architectures
Source: Int J Mol Sci. 2023 Jul 15;24(14):11521. doi: 10.3390/ijms241411521 (PMC10380299; doi:10.3390/ijms241411521)

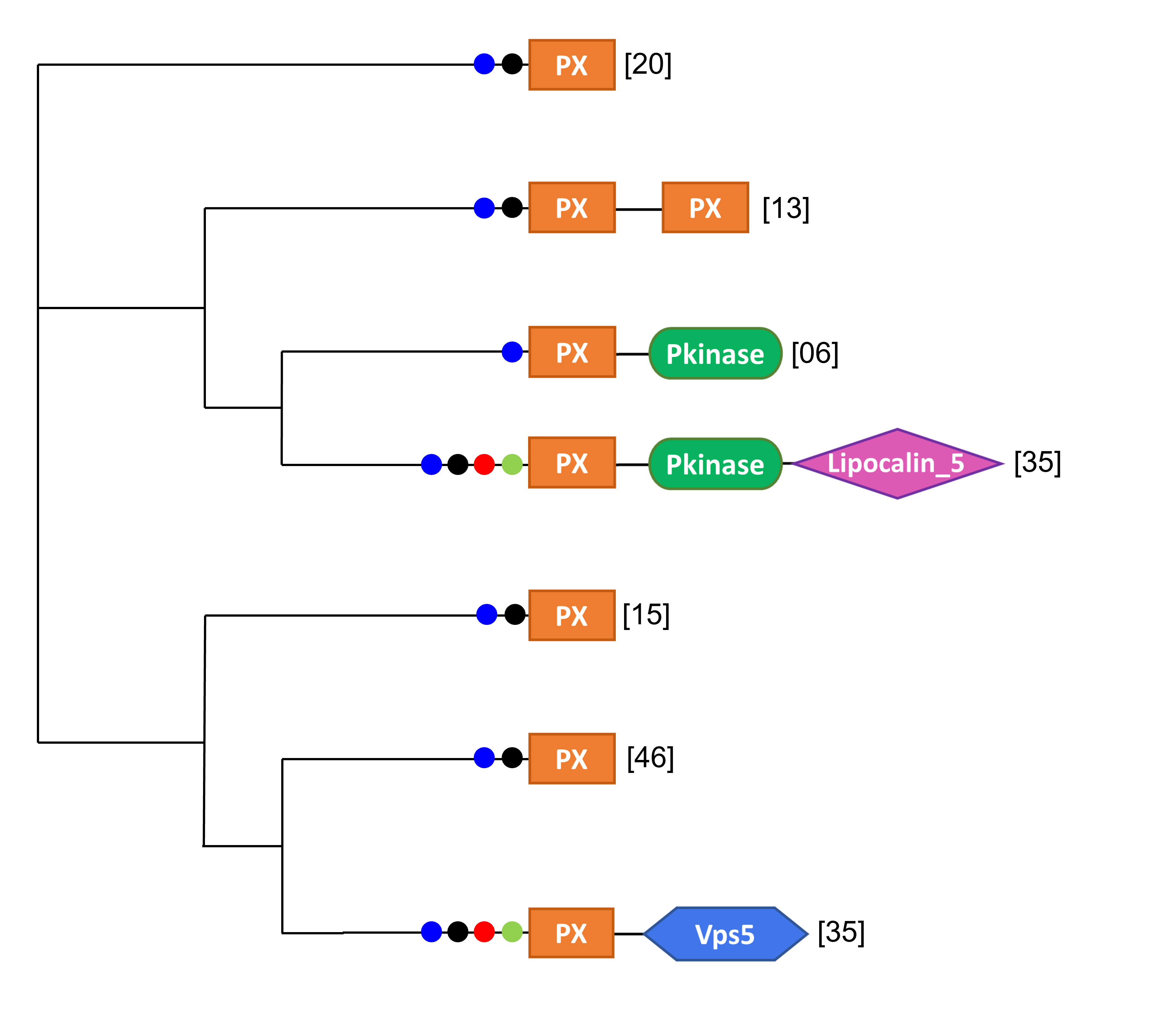

Supplement: Supplementary file 1 [file ijms-24-11521-s001.zip › Supplementary Figure S1.png]

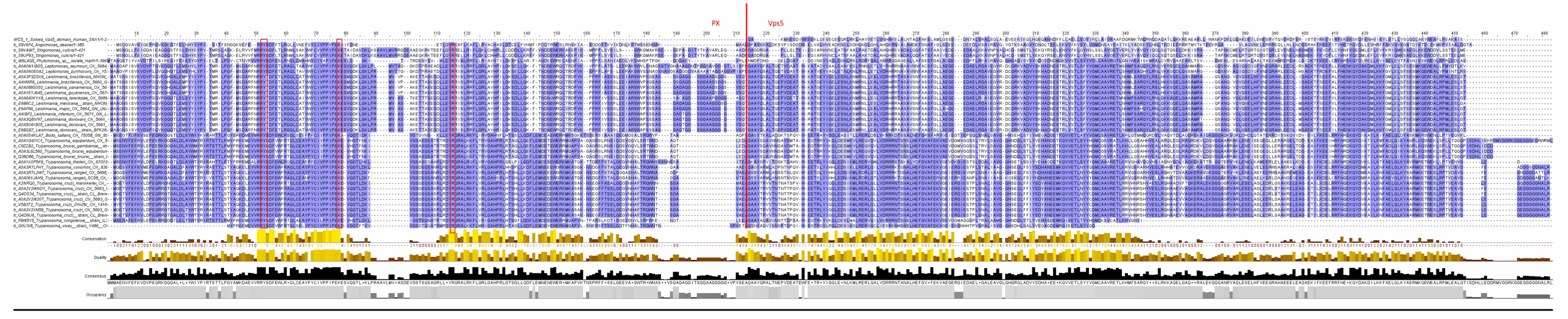

Supplement: Supplementary file 1 [file ijms-24-11521-s001.zip › Supplementary Figure S3.jpg]

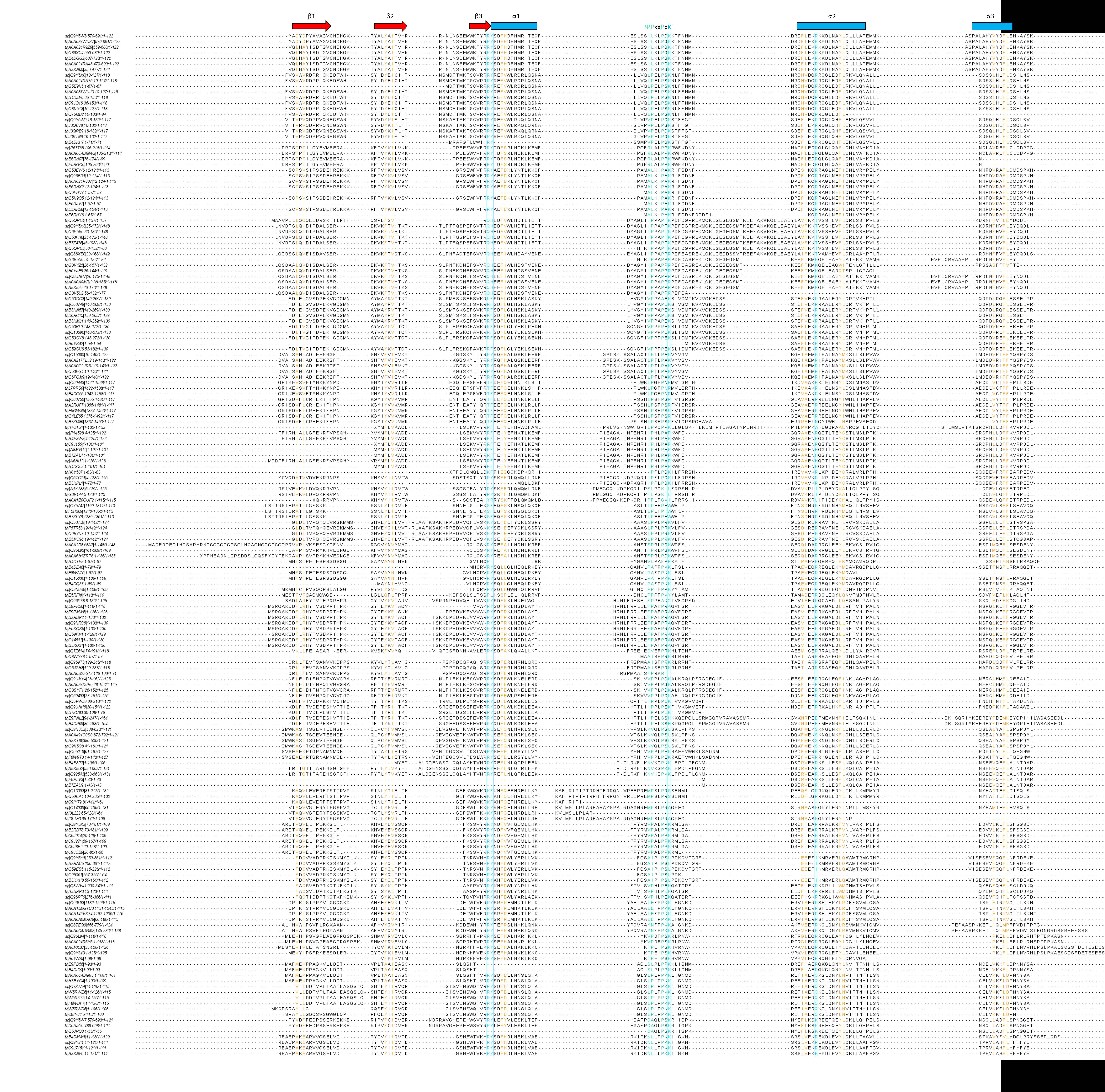

Supplement: Supplementary file 1 [file ijms-24-11521-s001.zip › Supplementary Figure S4.jpg]
